# Static Mechanical Properties of Expanded Polypropylene Crushable Foam

**DOI:** 10.3390/ma14020249

**Published:** 2021-01-06

**Authors:** Przemysław Rumianek, Tomasz Dobosz, Radosław Nowak, Piotr Dziewit, Andrzej Aromiński

**Affiliations:** 1 Faculty of Automotive and Construction Machinery Engineering, Warsaw University of Technology, 02-524 Warsaw, Poland; radoslaw.nowak@pw.edu.pl (R.N.); andrzej.arominski@pw.edu.pl (A.A.); 2 Faculty of Mechanical Engineering, Department of Machine and Vehicle Design and Research, Wroclaw University of Science and Technology, 50-370 Wrocław, Poland; tomasz.dobosz@pwr.edu.pl; 3 Faculty of Mechatronics and Aerospace, Military University of Technology, 00-908 Warsaw, Poland; piotr.dziewit@wat.edu.pl

**Keywords:** compressive deformation, EPP foam, foam, microstructure, strain rate

## Abstract

Closed-cell expanded polypropylene (EPP) foam is commonly used in car bumpers for the purpose of absorbing energy impacts. Characterization of the foam’s mechanical properties at varying strain rates is essential for selecting the proper material used as a protective structure in dynamic loading application. The aim of the study was to investigate the influence of loading strain rate, material density, and microstructure on compressive strength and energy absorption capacity for closed-cell polymeric foams. We performed quasi-static compressive strength tests with strain rates in the range of 0.2 to 25 mm/s, using a hydraulically controlled material testing system (MTS) for different foam densities in the range 20 g/dm^3^ to 220 g/dm^3^. The above tests were carried out as numerical simulation using ABAQUS software. The verification of the properties was carried out on the basis of experimental tests and simulations performed using the finite element method. The method of modelling the structure of the tested sample has an impact on the stress values. Experimental tests were performed for various loads and at various initial temperatures of the tested sample. We found that increasing both the strain rate of loading and foam density raised the compressive strength and energy absorption capacity. Increasing the ambient and tested sample temperature caused a decrease in compressive strength and energy absorption capacity. For the same foam density, differences in foam microstructures were causing differences in strength and energy absorption capacity when testing at the same loading strain rate. To sum up, tuning the microstructure of foams could be used to acquire desired global materials properties. Precise material description extends the possibility of using EPP foams in various applications.

## 1. Introduction

For designing protective structures or energy absorbing elements, the closed-cell material, such as expanded polypropylene (EPP), is the most reasonable choice. Energy-absorbing structures have the ability to take over the kinetic energy of impacts. This energy is equivalent to the work of force destroying the material (crushing, breaking). Closed-cell EPP foam is commonly used in automobile bumpers for absorbing impact energy. In this application, foam energy absorbing characteristics are related to the loading received by the bumper reinforcement beam and body frame. Varying foam density and thickness changes the energy-absorbing capabilities, enabling further optimization of bumper systems. Low production costs, low weight and high energy-absorbing capabilities enable foam sandwich structures to be used in aerospace and automotive industries. Foam materials are often manufactured as steam chest moulding, using a sintering-like process (heat and pressure). The final performance of manufactured parts may be affected by many variables such as: manufacturing method, gas used for closed cell foams, cell geometry, constituent material. Kinetic energy-absorbing capability on the right level is very important, because values of the peak force and acceleration over the threshold could cause injury or damage. Energy absorbed during single or multiple impacts can be described in several ways [1,2,3]. For example, as a relation between peak impact deceleration of the real foam and “ideal” foam, and the ability to completely absorb impact energy. A lot of empirical data is needed in order to assess this factor, as it is dependent for example on material thickness and impact energy. Alternatively, this factor can be evaluated by obtaining curves representing the absorbed energy as a function of peak stress. Such curves can be derived from stress–strain dependence. Research and analyses conducted by Rusch [4] lead to the construction of peak stress vs. specific energy absorption curves. This method still relies on empirical evaluation of foam characteristics at different levels of impact energy, but it provides better generality than the J-factor. Burgess et al. [5] and Castiglioni et al. [6] attempted to model foam cushioning behaviour. The so-called Maiti diagrams [7] are a more refined approach. In those diagrams, both normalized to foam modulus, energy per volume unit is plotted against stress level for different foam densities. Selecting proper foam material and density for particular application can be done by finding a set of points corresponding to densification onset for given foam density. An extensive data set acquired at different strain rates is needed to cover application requirements for particular material use. The material structure and the strain rate of polymer foams was considered by Luca Andena et al. They have used Nagy’s phenomenological model and determined the material stress–strain behaviour at a reference strain rate [3]. To predict foam behaviour under specific conditions and optimizing design of energy absorption devices, various models were developed, trying to capture actual material mechanics. The Gibson and Ashby [8] model is commonly used to gain basic understanding of foam behaviour. The Gibson and Ashby model assumes regular cell structure. Foams with irregular structures can be described by this model, but with limited predicting capabilities. Different models, such as Kelvin [9], Shulmeister [10] or Roberts-Garboczi [11,12] try to overcome limitations of the Gibson and Ashby model. All of the above models are unable to realistically represent real foam structure and all of them have limited accuracy. Mechanical properties and energy absorption capabilities of the foams change with temperature levels. The influence of temperature on foam characteristics was investigated in several studies [13,14,15]. According to Zhang et al. [16] EPP foam energy absorption capability of the component was found to be highly dependent on temperature [17].

The aim of this study was to investigate the impact of the structure of the material on energy dissipation in various conditions. The verification of the material properties was carried out on the basis of the results of experimental tests and simulations performed using the finite element method (FEM). The method of describing foam properties is used for analysis of protective elements in vehicles. Analyses take into account the foam materials in a manufacturing process, where we define: basic material and technologies (gas pressure used during foaming). Experimental tests were performed for various loads and at various initial temperatures of the tested sample. The presented method of material analysis allows for the selection of an appropriate hyper-elastic model and its modification, resulting in even better determination of material properties. During experimental tests, compressive stress test was performed with 10%, 60% and 80% relative deformation. Deformation values ware based on the methods specified in the standards [18,19].

Experimental, analytical and FEM simulation results were compared and presented in a graphical manner. FEM material model analysis takes into account the experimental results and modifications of the existing material models.

## 2. Materials and Methods

### 2.1. Experimental Methods

#### 2.1.1. Materials and Specimens

**Figure 1:**
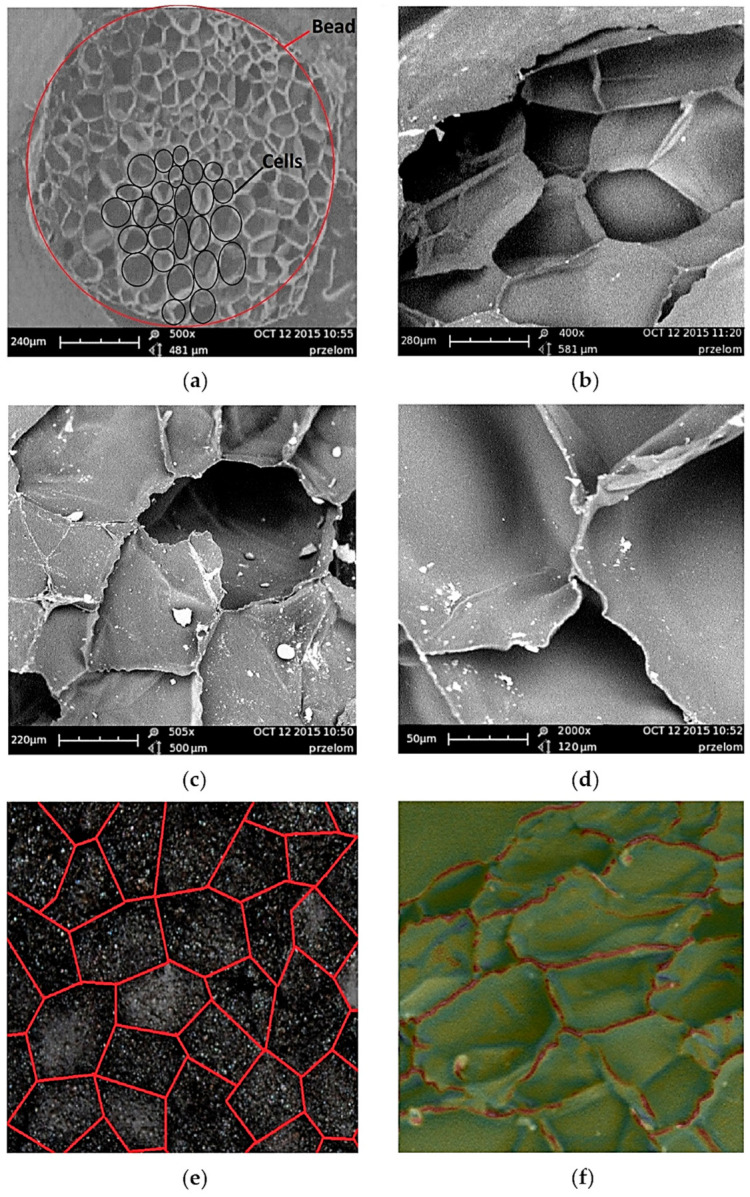
Scanning electron microscope (SEM) microphotographs cellular structure of expanded polypropylene (EPP) of density ρ = 20g/dm^3^ (**a**–**d**) microphotograph structure bead (**e**) diagram and photograph structure bead (**f**) morphology structure closed cell.

The material under investigation is a polypropylene polymer foam. EPP foam components are made from small beads, moulded into shape. At first polypropylene beads are expanded, forming larger, foamed beads with a diameter of 0.25–5 mm. The next stage is further bead expansion in a mould. At this stage the temperature is higher than in the first stage, causing melting EPP and sintering of the foamed beads into the final component. Process parameters of polypropylene expansion determine the degree of expansion, foam density and cellular structure. The complex cellular structure of each EPP bead and its interaction with adjoining beads allows any energy exerted in the executed part to be managed [17]. A typical cellular structure of an individual EPP foam bead is shown in Figure 1a–d. The research structure was determined using Phenom G2 scanning electron microscope (SEM) (Phenom-World BV, Eindhoven, The Netherlands).

The cross section images were taken by notching one side of a rectangular sample, cooling sample in liquid nitrogen and then breaking by bending while frozen. Breaking frozen samples was done to reduce potential plastic deformation in the breaking region of the sample. Then the samples were coated with gold using a sputter deposition method and imaged by a SEM device. Cellular structure can be seen in the inside of the single EPP bead. After extracting samples from the component, foam density was estimated by weighing specimens and measuring specimen dimensions using a calliper. The average density of samples taken from a component was 20 g/dm^3^ with a standard deviation of 0.5 g/dm^3^. Detailed structure tests were performed on rigid polypropylene foam of different densities 20 g/dm^3^, 30 g/dm^3^, 80 g/dm^3^, 120 g/dm^3^ and 200 g/dm^3^. 3D reconstruction software (Phenom ProSuite v2.3, Phenom-World BV, Eindhoven, The Netherlands) was used to measure structure of the selected areas. The software is based on the ProSuite system.

Due to the wide structural diversity of material (Figure 1e,f), which affect the mechanical properties, studies were carried out on averaged sizes of the closed cell of the materials, which is described by Equation (1). The mechanical tests for randomly selected cells show that for different foam densities, we obtain different values of stresses and strains, which can be seen in quasi-static compressive strength experiment tests.

**Figure 2:**
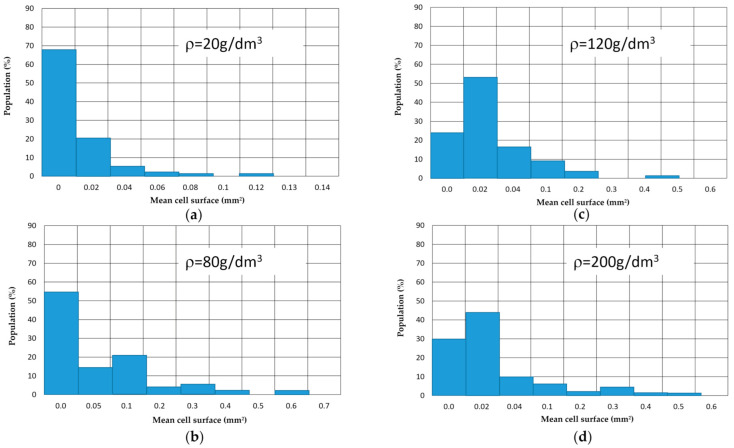
Representative measurement of average cell dimension (**a**) cell surface *ρ* = 20 g/dm^3^ (**b**) *ρ* = 80 g/dm^3^ (**c**) *ρ* = 120 g/dm^3^ (**d**) *ρ* = 2000 g/dm^3^.

The closed cell size test method is already used for the analysis of metallic foams [20,21,22,23]. The individual closed cell size is presented as *D_m_* [20,24]. More than 100 cells from each sample were selected for size calculation. The mean cell size *D_m_* is determined by the following equation *D_m_*,

(1) $$D_{m}=\frac{S_{i}}{\sum_{i=1}^{n}S_{i}}\times \sqrt{\frac{S_{i}}{\pi }}$$

where *n* is the total cell number, *S_i_* is the area of the *i* th pore, which was captured by image processing software (Phenom ProSuite v2.3, Phenom-World BV, Eindhoven, The Netherlands) [22]. Figure 2 shows a representative measurement of average cell dimension.

**Figure 3:**
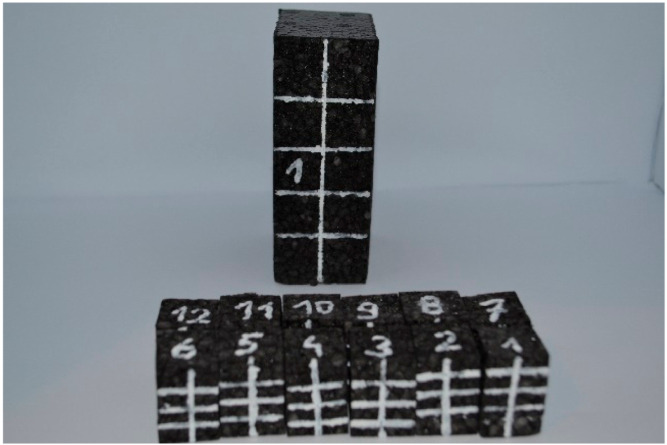
Tested specimens EPP foam.

Due to the requirements of the test machines used for research, samples were prepared in two sizes: 80 mm × 80 mm, 40 mm height and 20 mm × 20 mm, 30 mm height. Samples were prepared from foams with a density range from 20 g/dm^3^ to 220 g/dm^3^ and initial dimensions of moulded rectangular blocks (500 mm × 800 mm × 80 mm), which was made by automotive EPP manufacture (Izoblok, Chorzów, Poland). The specimens used for compression tests are shown in Figure 3.

**Figure 4:**
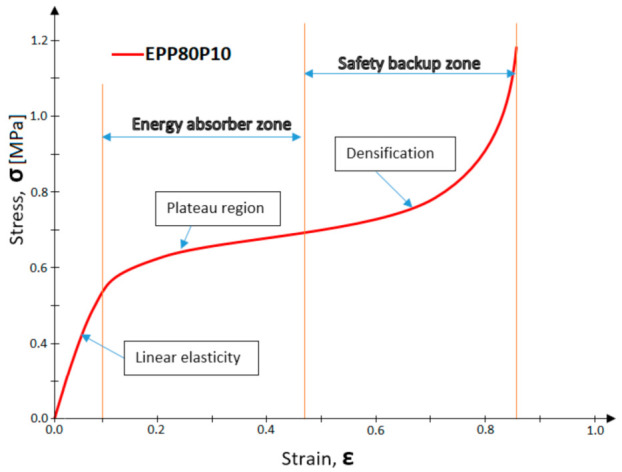
Regions of EPP static stress-strain curve.

Tests were performed on samples of different sizes, which allowed the estimate to influence the stress–strain of a tested material [25,26]. Tests using big and small samples were performed because of the needs of the automotive industry, where the energy absorption elements with cuboidal shape have a wide range of cross-section areas. These samples were made in various sizes in order to understand the geometric effect [27]. Experimental compression tests were performed at different strain rates on identical samples to allow direct comparison of quasi-static responses [28]. Exact dimensions were measured using a calliper prior to each test. A typical static stress–strain curve, with each region identified, for EPP foam is presented in Figure 4.

A typical stress–strain curve contains linear elasticity and densification regions. The area between those regions is characterized by a slowly rising plateau. From 0% to 5–10% strain the material is in the linear elasticity region, which defines foam Young’s modulus at specific density. The plateau region refers to the material’s elastoplastic behavior. In this region, cellular structures dissipate the applied load by transferring energy through the cellular structures of both individual beads and between individual beads. Moulded EPP has an anisotropic nature, this enables it to transfer energy in the most efficient manner giving EPP foam superior resilience. Due to this, the stress–strain curve can be maintained after repeated impacts and at quasi-static test speeds (<25 mm/s). When all cells have collapsed, the densification area is reached.

#### 2.1.2. Quasi-Static Test

**Figure 5:**
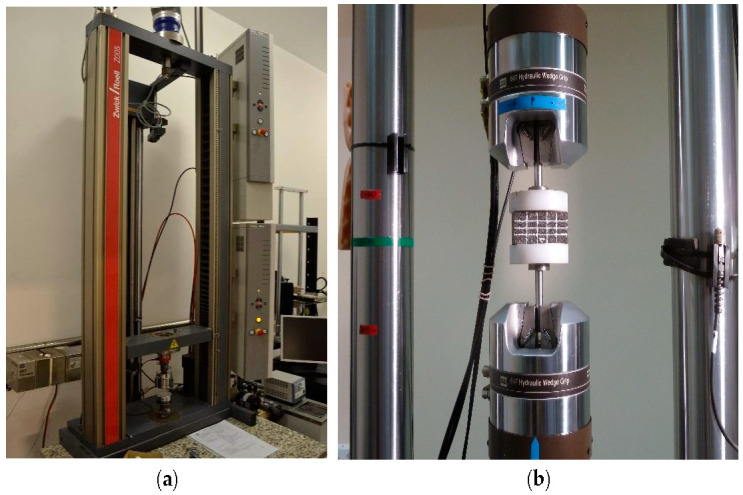
Testing machines (**a**) Zwick/Roell Z005, (**b**) Q-test 10 material testing system (MTS).

Compressive strength tests of foam samples were conducted using a testing machine located in the laboratory of the Institute of Machine Design Fundamentals at the Faculty of Automotive and Construction Machinery Engineering at Warsaw University of Technology and Department of Materials Science and Engineering at Warsaw University of Technology. Compressive strength was determined using Q-test 10 material testing system (MTS Systems GmbH, Berlin, Germany) and Zwick/Roell Z005 testing machines (ZwickRoell GmbH & Co., Ulm, Germany) (Figure 5). Compression force between static and moving pistons was recorded during the tests. This was the first part of the experimental testing of samples. Graphs showing dependence between stress and strain were prepared based on acquired data. Samples were prepared in the form of cuboids using foams of different density, varying from 20 g/dm^3^ to 220 g/dm^3^. Table 1 shows the static strength of used materials delivered by the material manufacturer.

**Figure 6:**
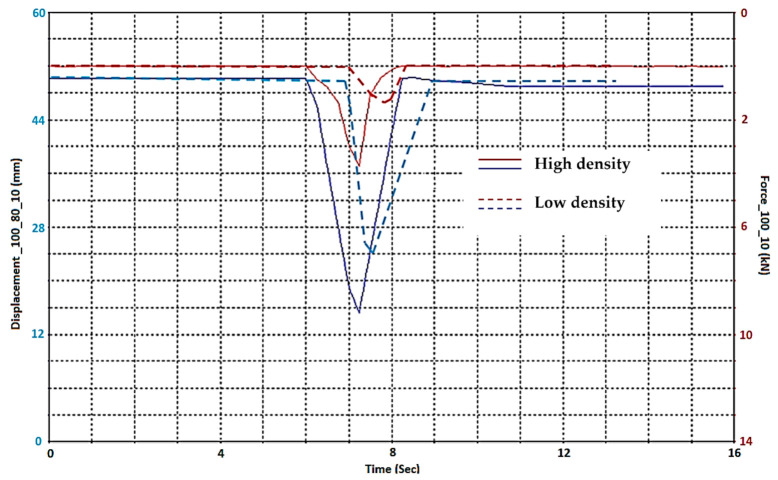
Actuator position vs. time for different sample densities, low (20 g/dm^3^) and high (200 g/dm^3^) density EPP foams.

Research was conducted on samples with temperatures of −30 °C, 23 °C, 80 °C. The environmental chamber was used to keep the appropriate and constant temperature level in a single test and, of course, this facilitated an easy change to the temperature value, thus it was possible to test the influence of the aforementioned parameters. A pyrometer was used for control of temperature measurement during the tests. The strain rate compression test includes uniaxial compression of tested samples at variable loading rates on cube specimens. Actuator position vs. time for different sample densities is shown on Figure 6. Velocity decreases with time, which corresponds to changes in strain rate from 0.2 to 25 mm/s throughout the test.

**Figure 7:**
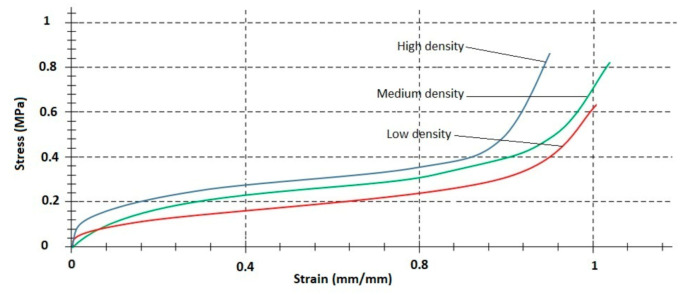
Comparison strain of representative stress–strain curves at strain rate 25 mm/s for low (20 g/dm^3^), medium (80 g/dm^3^) and high (200 g/dm^3^) density EPP foams.

The stress–strain curve is perceived to be dependent on foam density. Tests were repeated three times, observed spread in obtained test curves was minimal for all tested foam densities. Figure 7 shows three samples of different densities low-, medium- and high-comparison strain of representative stress at strain rate 25 mm/s.

**Figure 8:**
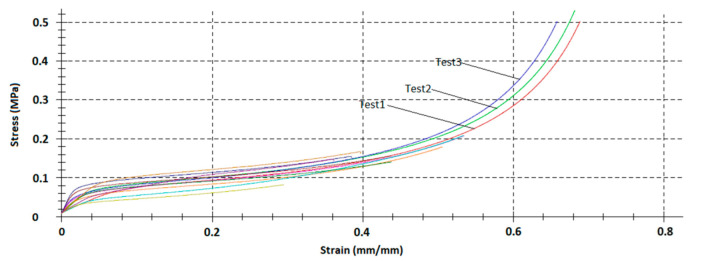
Varying strain rate compression tests three repeats (Tests 1–3) for low-density (20 g/dm^3^) EPP foams, sample size 20 mm × 20 mm, 30 mm height.

Figure 8 shows three repeated varying strain rate compression tests for low-density EPP foam.

### 2.2. Numerical Implementation

The finite element method (FEM) was used for model calculations. Numerical simulations of the EPP foam were performed in Abaqus software version 6.12 (Dassault Systemes, Vélizy-Villacoublay, France). Foam was appropriately modelled for numerical analysis. Simulations were performed using Abaqus/Standard and Abaqus/Explicit module as the incorporated thermo-mechanical task (due to temperature changes and associated changes in stress).

**Figure 9:**
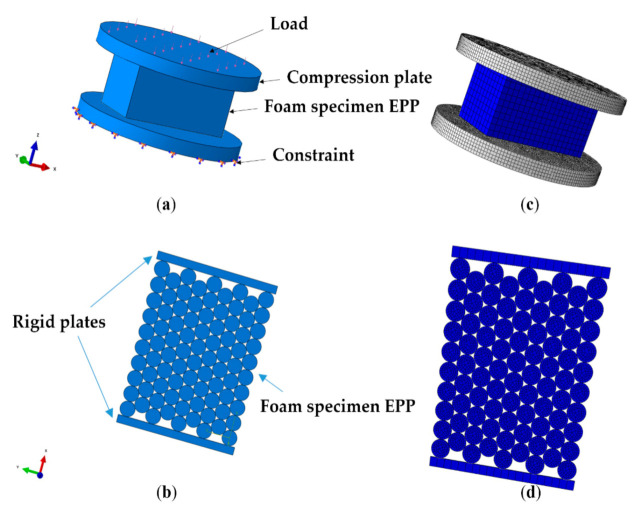
Uniaxial compression EPP specimen (**a**) CAD model (**b**) structure small beads: section view of CAD model; (**c**) finite element model of specimen; (**d**) structure small beads: section view of Finite element model.

Samples of the same dimensions as in the experimental tests were used for simulation of the compression tests. Stress–strain curves were used to estimate levels of correlation. In the simulations, FEM foam specimens were compressed by modeling a rigid compression steel plate. Plate velocity during the test was specified as the same as the experiment by using the velocity vs. time curve and it is dependent on a required strain rate to be simulated. Contact between the compression plate and the tested specimen was defined as automatic, with general constrain formulation [29]. As shown on Figure 9a, one of the nodes of the specimen was fully constrained to simulate rigid substrate.

Simulation analysis allowed investigation of the phenomena occurring during deformation of the foam structure–tightening pores (separately for open and closed pores) in hyperelastic materials. Additionally, a specific issue that was considered was friction in the fixed joints, traditionally named construction friction, widely regarded as the contact task, taking into account making connection and workloads. The finite element method was used for validation and prediction of the developed material models capabilities during complex load cases.

While describing a material’s properties, all of the relevant assumptions for this kind of material was used: theory of hyper-elastic materials [30], model of elastic foam, elastic-plastic and plastic foam and other causes of energy dissipation–internal dampening and material friction. Simulations were carried out using models for crushable foam. The volumetric hardening model assumes that the hydrostatic compression strength evolves as a result of compaction or dilation [29]. Simulations were performed in a simplified system (modeled the plate without fixing bars). Table 2 shows material and element properties of the numerical simulations of the small sample.

The structures were also modelled as small beads Figure 9b making it possible to introduce various degrees of structural irregularity.

The mesh was created at the part level, since all parts used in this investigation are dependent. Figure 9c shows a finite element model of specimen and Figure 9d shows a section view of finite element model of the structure of small beads.

In the numerical simulation the temperatures of the analyzed samples were defined [29]. Temperatures were adopted for the tested samples −30 °C, 23 °C and 80 °C. Numerical analysis was carried out in the field of static calculations in strain rate from 0.2 to 25 mm/s throughout the test.

## 3. Results

### 3.1. Experiment

**Figure 10:**
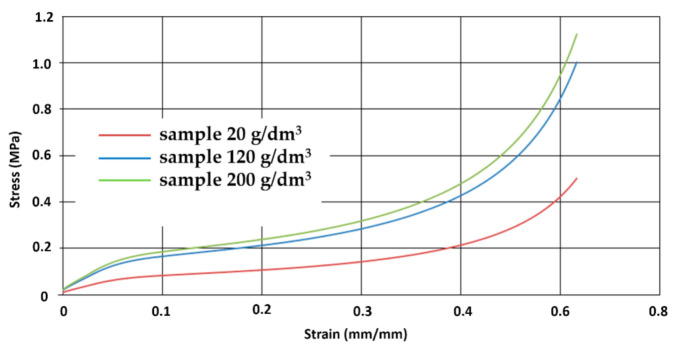
The stress–strain curves of three kinds of EPP foam at a quasi-static loading rate of 2 mm/s.

In Figure 10 the yield curve for quasistatic compression for three types of samples that have different densities but the same dimensions can be seen. During the tests, it was observed that the stress value becomes higher with the increase of density.

**Figure 11:**
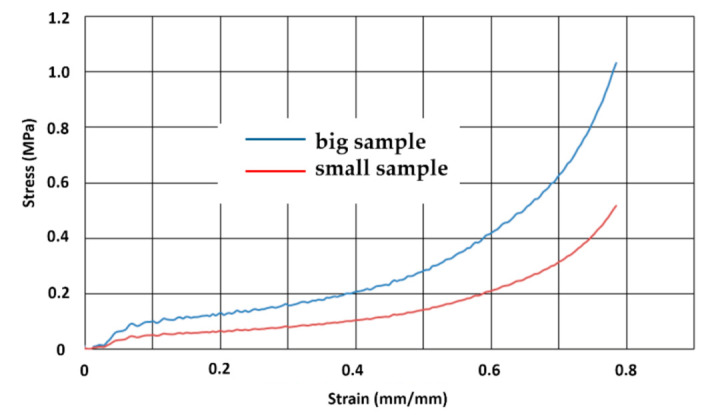
The stress–strain curves of big (80 mm × 80 mm, 40 mm height) and small (20 mm × 20 mm, 30 mm height) sample of EPP foam at a quasistatic loading strain rate of 2 mm/s.

It can be observed that a higher unit weight of the granules and a higher packing index in a high-density sample resulted in a high modulus of elasticity. Due to the lower strain rate, the material strengthening phenomenon resulting from gas compression in cavities is negligible. Figure 11 shows the yield curves for quasistatic compression tests of samples of various shapes.

**Figure 12:**
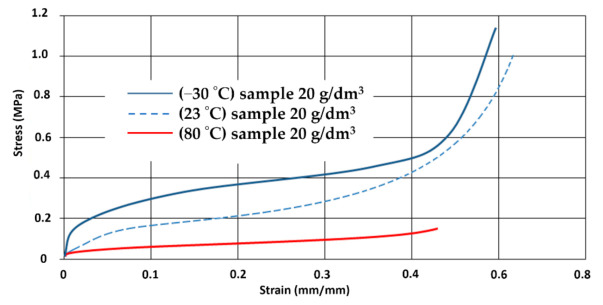
The stress–strain curves of EPP foam (density 20 g/dm^3^) at a quasistatic loading, strain rate of 2 mm/s and different temperature, sample size 20 mm × 20 mm, 30 mm height.

The research showed a significant influence of temperature on the samples tested. This has been confirmed by determining the Young’s modulus in EPP materials. With increasing temperature, stresses significantly decrease, the material loses its elastic properties. Figure 12 shows the differences in stresses and strains depending on the temperature of a sample of the same density. The sample at 80 degrees was damaged with a strain value of 0.46.

**Figure 13:**
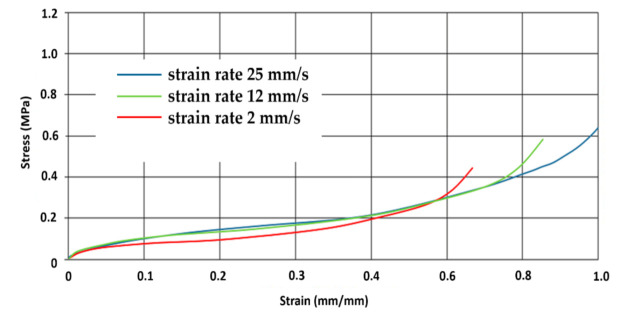
The stress–strain curves of EPP foam (density 20 g/dm^3^) at a quasistatic loading, different strain rate.

Figure 13 shows the differences in stress and strain depending on the quasistatic loading of the different strain rate in the sample of 20 g/dm^3^.

### 3.2. Numerical Simulations

**Figure 14:**
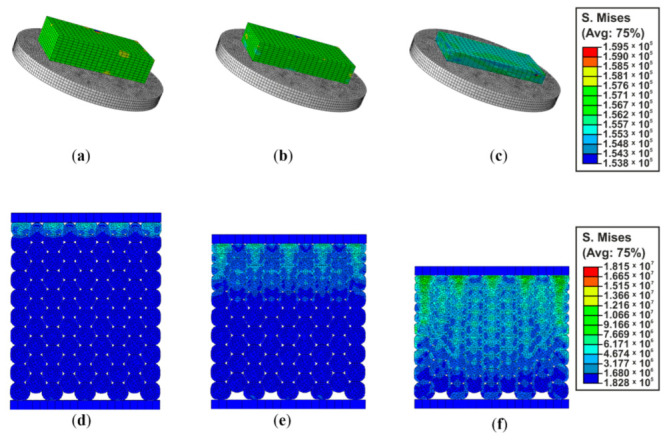
Reduced stresses of relative deformation at (**a**) 10% (**b**) 60% (**c**) 80% (**d**) 10% (**e**) 30% (**f**) 60%.

Due to the presence of large volumetric deformations considered in this work, there is the need for description of the strength other than the most commonly used Huber–Mises–Hencky model of plasticity condition. The model used is called “crumbling foam” (crushable foam). Conducted research and analysis have shown that elastic deformation occurred up to 60% load, over 60% there were elasto-plastic deformations. The examples of reduced stress results for one of the simulations in the Abaqus program depending on the compression are shown in Figure 14a–c.

To investigate a structure of the material simulations of interconnected EPP granules were carried the detailed influence of the cellular material structure. Figure 14d–f shows examples of reduced stress of the sample.

**Figure 15:**
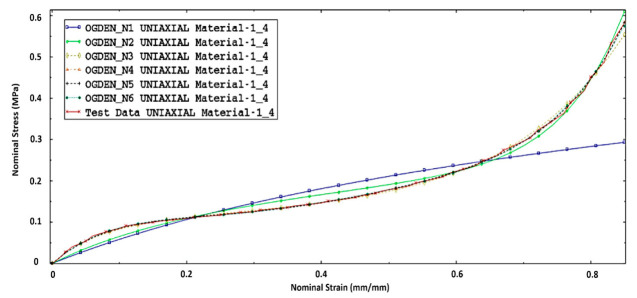
The simulations nominal stress-nominal strain curves of the Ogden model and experimental test data at a quasistatic loading rate of 2 mm/s.

Simulation analysis of different material models was conducted. By comparing them to the experimental studies, we have been able to make a series of comparisons for different types of models. Except for the values of the coefficient *α_i_* (*α_i_* represents the shape of the yield ellipse in the stress plane and can be calculated using the initial yield stress in uniaxial compression [29]) others have led to a non-linear model, which allows for the description of materials and compressibility. The values of the coefficients were determined by approximation based on experimentally defined stress–strain. When using the FEM application, hyperelastic models can be chosen, for which the set of properties have been described. The correct choice of the applied material model should be completed with the comparison of the results of the FE model and experiment. In the case of acceptance of the description of the coefficients *α_i_* = 2, 4, 6,..., then we have a polynomial model, including various special cases: the models of Mooney–Rivlin [31,32] and Neo-Hookean [33]. Figure 15 shows a series of compression curves set experimentally by Ogden models for foams with a density of 20 g/dm^3^.

By introducing the equation of *α_i_* coefficients with fractional values, non-linear models could be obtained already in the first approximation. Final results of the process revealed that the consistent stresses waveforms in the real and numerical studies have occurred for the Ogden model. The material has been described by the third row of Ogden’s model [29,34]. According to simulation studies, Ogden’s model claims that resilience potential can be described as:

(2) $$U=\sum_{i=1}^{N}\frac{2\mu_{i}}{\alpha_{i}^{2}}\left({{\overset{-}{\lambda }}_{1}}^{\alpha_{i}}+{{\overset{-}{\lambda }}_{2}}^{\alpha_{i}}+{{\overset{-}{\lambda }}_{1}}^{\alpha_{i}}-3\right)+\sum_{i=1}^{N}\frac{1}{D_{i}}{\left(J_{el}-1\right)}^{2i}$$

where:

(3) $${\overset{-}{\lambda }}_{i}=J^{-\frac{1}{3}}\lambda_{i}\to {\overset{-}{\lambda }}_{1}{\overset{-}{\lambda }}_{2}{\overset{-}{\lambda }}_{3}=1$$

$\lambda_{1}{}^{\alpha_{i}}$—invariant of deformation state; *J_el_*—sample volume change dependent parameter, *µ_i_*, *α_i_*, *D_i_*—experimentally determined coefficients.

Hence, the first part of Ogden’s strain energy function depends only on ${\bar{I}}_{1}$ and ${\bar{I}}_{2}$. Ogden’s energy function cannot be written explicitly in terms of ${\bar{I}}_{1}$ and ${\bar{I}}_{2}$. It is, however, possible to obtain closed-form expressions for the derivatives of *U* with respect to ${\bar{I}}_{1}$ and ${\bar{I}}_{2}$.

The value of N and tables giving the values as functions of temperature are specified by the user.

In the Ogden form the initial shear modulus, $\mu_{o}$, depends on all coefficients:

(4) $$\mu_{o}=\sum_{i=1}^{N}\mu_{i}$$

(5) $$k_{o}=\sum_{i=1}^{N}2(\frac{1}{3}+D_{i})\mu_{i},$$

and the initial bulk modulus, $k_{o}$, depends on $D_{i}$ as before. The user can request that Abaqus calculate the $\mu_{i}$ and $\alpha_{i}$ values from measurements of nominal stress and strain.

(6) $$\sigma_{i}=2{\left(\lambda_{i}\right)}^{-1}\sum_{i=1}^{N}\frac{\mu_{i}}{\alpha_{i}}\left(\lambda_{i}{}^{\alpha_{i}}-J^{-\alpha_{i}D_{i}}\right),$$

where the material parameters *µ_i_*, *α_i_*, and $\;D_{i}$ (*i* = 1, 2, 3) can be determined by fitting the experimental nominal stress–strain curve.

The coefficients of Ogden’s material model, used in the numeric analysis FEM, are presented in Table 3.

For modeling foam, the Ogden model was modified with the introduction of the real exponent in the second part of the equation that describes the volume deformations (in this case, also non-linear dependencies), Table 4.

## 4. Conclusions

Tests were conducted to characterize the material structure and the mechanical response of EPP foam. During the research on the structure, SEM photographs showed that depending on the density of the foam there are finer cell structures with smaller cells. Measurements of compression and responses from the foam were presented at different speeds of deformation. Samples were tested in compression tests with strain rates in the range of 0.2 to 25 mm/s. Convergence of results for different strain rates conditions implies no gas strengthening. The research for samples having different temperatures show large effects of high temperatures on the test material. The material reach the yield point and the sample was damaged. A huge influence was observed on the shape due to stresses and strains. There were noticeable differences in the mechanical responses between the foams of similar density as confirmed by the lack of reproducibility of the structure. The main idea of this work was to identify and propose types of hyperelastic models for specific groups of material, which are used for increasing safety levels in the automotive industry. The analysis performed allowed us to extend the possibilities of using modern construction materials, plastics and composites. It is impossible to perform such analysis without precise material description. Usage of known hyperelastic material models–polynomial, Ogden’s normal and reduced non-linear allowed us to describe the properties of the tested foam material (EPP) correctly. The use of the modified Ogden’s model makes it possible to accurately determine the description of the material, which makes it possible to increase the accuracy and effectiveness of the simulation. The research carried out allows an even better selection of the material and its properties.

## Figures and Tables

**Table 1:** Material properties of the specimens.

Specimen	*ρ*_1_	*ρ*_2_	*ρ*_3_	*ρ*_4_	*ρ*_5_
Density (g/dm^3^)	20	30	80	120	200
Strength (10^−2^ MPa)	23.52	42.14	95.4	98	110
Failure strain (%)	12	17	16	30	35
Compressive plastic strain (%)	8.5	8.6	9.2	11	16

**Table 2:** Material and element properties for the model used in the numerical simulations.

Density of Foam EPP (g/dm^3^)	Elastic Modules E (MPa)	Poisson Ratio υ	Tested Elements	Mesh Element Size (m)	Mesh Refinement	Coefficient of Static Friction	Coefficient of Kinetic Friction	Coefficient of Decay
20	3.5	0	C3D8R, CPS4R	0.002	2000	0.6	0.5	0.1
30	4.3	0.2	C3D8R, CPS4R	0.002	2000	0.6	0.5	0.1
80	8.2	0.3	C3D8R, CPS4R	0.002	2000	0.6	0.5	0.1
120	16.0	0.3	C3D8R, CPS4R	0.001	16,000	0.6	0.5	0.1
200	92.1	0.3	C3D8R, CPS4R	0.001	16,000	0.6	0.5	0.1

**Table 3:** Material properties of the specimens. Coefficients of Ogden’s model.

*i*	*μ_i_*	*α_i_*	*D_i_*
1	−1,062,310.500	7.7010	8.6448
2	781,324.564	8.0848	0
3	776,736.530	−11.4395	0

**Table 4:** Material properties of the specimens modify the coefficients of Ogden’s model.

*i*	*μ_i_*	*α_i_*	*D_i_*
1	−832,131.490	16.209480	8.935334
2	831,230.727	16.211660	0
3	−2,528,968.430	−1.172925	0

## Data Availability

Data sharing not applicable. No new data were created or analyzed in this study. Data sharing is not applicable to this article.
